# Dataset of stopwords extracted from Uzbek texts

**DOI:** 10.1016/j.dib.2022.108351

**Published:** 2022-06-03

**Authors:** Khabibulla Madatov, Shukurla Bekchanov, Jernej Vičič

**Affiliations:** a Urgench state university, 14, Kh. Alimdjan str, Urgench city, 220100, Uzbekistan; b Research Centre of the Slovenian Academy of Sciences and Arts, The Fran Ramovš Institute, Novi trg 2, 1000 Ljubljana, Slovenija; c University of Primorska, FAMNIT, Glagoljaska 8, 6000 Koper, Slovenia

**Keywords:** Stop words, Machine Learning, Unigram, Bigram, Collocation

## Abstract

Filtering stop words is an important task when processing text queries to search for information in large data sets. It enables a reduction of the search space without losing the semantic meaning. The stop words, which have only grammatical roles and not contributing to information content still add up to the complexity of the query. Existing mathematical models that are used to tackle this problem are not suitable for all families of natural languages [1]. For example, they do not cover families of languages to which Uzbek can be included. In the present work, the collocation method of this problem is o ered for families of languages that include the Uzbek language as well. This method concerns the so-called agglutinative languages, in which the task of recognizing stop words is much more difficult, since the stop words are “masked” in the text. In this work the unigram, the bigram and the collocation methods are applied to the “School corpus” that corresponds to the type of languages being studied.

## Specifications Table

**Table:** 

Subject	Computer Science Applications
Specific subject area	Stopword lists creation for Uzbek language.
Type of data	Table
How data were acquired	Data were acquired using three methods presented in this paper. The methods were used on a newly constructed corpus called: “School corpus”.
Data format	Raw
Parameters for data collection	The data gathering was organized using the collected corpus and ad-hoc implementation of all three methods presented in the remainder of the paper.
Description of data collection	A corpus of Uzbek language (called “School corpus”) was constructed using online study materials comprising of 731,156 words, of which 47,165 are unique words. Three lists of stopwords were constructed using methods presented in Sections 1.2, 1.3, 1.4. The methods are based on the popular machine learning algorithm TF-IDF and augmented with some simple linguistic knowledge. The software implementing the presented methods was written ad-hoc during the experiment.
Data source location	Urgench – Uzbekistan
Data accessibility	Repository name: Zenodo
	Data identification number: 10.5281/zenodo.6319953
	Direct URL to data: https://doi.org/10.5281/zenodo.6319953
	The lists were extracted from a corpus of school texts. The texts are freely available on the national book web portal: https://kitob.uz/.
	The corpus can be re-constructed using the list of urls of all files comprised in the corpus.
	The list is part of the dataset (list_of_urls_of_school_corpus.txt).
	Instructions for accessing these data: unzip the archive, all data is distributed in three files: stopwords_unigrams.txt, stopwords_bigrams.txt, stopwords_bigrams_with_collocations.txt. Data is distributed in simple text format, each line represents one enumerated stopword.

## Value of the Data

The database of stop words obtained by the authors of the article is useful for searching and removing stop words from texts in the Uzbek language and is of great importance for the following processes:

– Sentiment analysis of texts;
– Clustering texts into classes;
– Information retrieval on texts;
– Text Mining and etc.

These are the functionalities and users that can most benefit from the presented data:

– Developers of online search engines, online catalogues (the searches will be more concise and faster if first eliminating stopwords),
– Developers of automatic information retrieval systems (by removing the stopwords from unstructured text, the reduced search-space greatly simplifies the IR process, both in quality and speed),
– Researchers and developers working on sentimental analysis of text, again the re-duced text size is easier to manage.

The usage is pretty simple: the stopwords are gathered in three files, the first one for monograms and the consequent two for bigrams. All three files have a simple structure: a stopword per line, preceded by the incremental ID number. The stopwords can be used as simple lookup dictionaries in order to detect and eliminate the stopwords from the observed texts.

## 1 Data Description

Removing stop words from a text is the process, in which the text is transformed to semantically equivalent text, that is represented by fewer words. As a result, instead of analyzing the original, we move on to a shorter, more concise, convenient text that is equivalent in meaning. Of course, when a computer works with compact text, it works faster than when working with a large text (the search space is smaller).

There has been a lot of research already done in the task of stopword detection. Most of the recent literature presents methods that are based on statistical approaches. The sources mainly use the TF-IDF method to analyze the text, such as [2], [3], [4], [5], [6], [7]. In a wide group of languages, the stopwords may be simply exposed by their relatively higher occurrence frequencies. But, in agglutinative or inflectional languages, a stopword may be observed in several different surface forms due to the inflection producing a higher number of possible candidates, including false positives. There has been some research involving the search for stopwords for agglutinative languages, such as [5,8], and [7].

Three lists of stopwords were extracted from the School corpus, presented in Section 1, comprising the first comprehensive archive of stop words of Uzbek language. Each list was constructed using a different method, named Unigram, Bigram, and Collocation and presented in Sections 1.2, 1.3, 1.4. By the definition of the output (only single words), the Unigram method list has no intersection with the remaining two lists, the Unigram and Collocation lists have an overlap, being constructed from word pairs with similar properties. The downside of our stopwords list is that in the collection process same letters were simplified, such as the letters -o, -g instead of -o ’, -g’, although a list of stop words with “-g’” were manually added. The presented stopwords lists are most suitable for extracting annotation from the text.

Some basic information about the files is presented in Table 1, the lists are available at Zenodo with DOI:

Lists of Uzbek stopwords: https://doi.org/10.5281/zenodo.6483960

**Table 1:** The three lists of stopwords, unigrams – contains single words that are stopwords, bigrams – contains pairs of words detected by the bigram method, collocations – contains pairs of words detected by the collocation method.

File name	Nr. of entries
stopwords unigrams.txt	2,357
stopwords bigrams.txt	4,547
stopwords bigrams with collocations.txt	24,489

## 2 Experimental Design, Materials and Methods

The development of spoken language starts at home in the local environment, but the school plays a key role in the development of human thinking. Therefore, it is a natural way to start studying the automatic analysis of texts from school textbooks. The corpus consists of 25 school textbooks available at the Uzbek book portal^1^ and was constructed for the problem of automatic detection of stop words in the form of the “School corpus”. The corpus can be used for other research purposes ranging from comparative linguistics to natural language processing. The corpus can be re-constructed using the list of URLs that are stored in the dataset in the file: list_of_urls_of_school_corpus.txt.

Words that when removed from the text, do not affect the meaning of the text, are called stop words.

### 2.1 Methods overview

This section presents an overview of existing methods and available data including a comparison of the presented data and methods. Multi-Class Text Classification of Uzbek News Articles using Machine Learning is presented in [9]. The paper focuses on the text classification of Uzbek News articles. Stop words are one stage of its algorithm. Although the paper presents a method for extracting stop words, the authors of this paper are limited only on translating the stop words from the English language. A non-negligible number of stopwords are translations of several English preposition words. As an example the 81^st^ word -dan where the English translation of this word is from. But this word does not exist in Uzbek language. A small list of stopwords in the Uzbek language can be found on the internet at the following address: https://groups.google.com/g/nltk-dev/c/6OkH_B6Nmcg. This list comprises only one-word stopwords and most of these words were extracted by our first method and are present in the first list in our dataset. Madatov et al. [10] present an overview of the methods that can be used to extract stopwords for the Uzbek language. Our methods are an extension of the presented methods and the dataset is the result of the presented methods. Uzbek News Categorization using Word Embeddings and Convolutional Neural Networks is presented in [11]. In this paper, the authors claim that they extended an already available list of Uzbek stop words. The method is presented, but the list is not available. The list of unigram stopwords was manually checked by an expert and also the nature of the detection process guarantees a low number of false positives (words in the list that are errors), the additional two lists have been constructed using statistical methods and should be treated as a list of potential stopword candidates. Ac-cording to Central limit theorem of Lyapunov [12] approximately 5% of words with(where TF-IDF is equal to or close to zero) can be stopwords. The actual accuracy of the presented methods for two bigram stopwords lists still needs to be investigated.

### 2.2 Unigram method

The considered “School corpus” consists of 731,156 words, of which 47,165 are unique words. The TF-IDF – term frequency–inverse document frequency [13] method was used to automatically detect stopwords. To do this, for each of 47,165 unique words, its frequency was determined (the number of occurrences in the texts of the School Corpus), and the inverse document frequency IDF(word) = ln(n/m) where n = 25 – number of documents and m is the number of documents, containing the unique word among 25 documents. By multiplying these two values we get 47,165 products (TF-IDF values). In the Uzbek language stopwords belong to one of the following word classes: pronoun, modal verb, particle, part of a rhyme, conjunction, introductory word, adverb, auxiliary word. Only words belonging to these classes were observed as candidates for stopwords. Roughly 5 percent of the products were close to 0 and the words corresponding to these products were declared as stop words. As a result, 2348 words are defined as stopwords. All these words were checked by an expert confirming the result. Although there were a negligible number of errors in the stopwords list, most of the list was confirmed by an expert. The result is not surprising as the list was constructed with a set of rules. The only errors were present by the errors in tagging. The Unigram stopwords are stored in the file stopwords_unigrams.txt, part of the file is presented in Table 2.

**Table 2:** Part of the Uzbek unigram stopwords list, each line starts with the enumerator, following a blank space delimiter and the stopword. The English translations of the presented excerpt are: hisobidan - by account, hosil – yield, hozir – now, ichida – within.

Nr.	stopword
…	…
70.	hisobidan
71.	hosil
72.	hozir
73.	ichida
…	…

### 2.3 Bigram method

In the Uzbek language, some words in the text are not considered stopwords when observed separately but can become stopwords when considered as collocation words. Here are a few examples to support our claim. Removing underlined words in the first sentences has almost no effect on the meaning of the sentences. In Example 1.1 when observed as individual words the words “turli” and “sohalardagi” are not stopwords, but if they are observed as a collocation, they become stop words.

Example 1.1 Konferentsiyada turli sohalardagi muhandislar ishtirok etishadi. (Engineers from di erent fields attend the conference).

Konferentsiyada muhandislar ishtirok etishadi. (Engineers attend the conference).

Example 1.2 Xalqimiz bir martalik shprits vositasida emlanadi. (Our people are vaccinated with a disposable syringe.)

Xalqimiz shprits vositasida emlanadi (Our people are vaccinated with a syringe.)

In Example 1.2, when viewed as individual words the words “bir” and “martalik” are not stopwords, but if they are observed as a collocation, they become stopwords. In the Examples 1.1 and 1.2, the words “turli sohalardagi (form the di erent fields)” and “bir martalik (disposable)” are pronouns. This means that searching for stopwords in the form of collocation words is important in automatic stopwords detection. For the school corpus, 731,155 pairs of words are considered. Of these, 489,857 are unique word pairs. The algorithm used for the automatic stopword extraction using Bigram method is as follows:

In a pair of words AB, let A be the first word and B be the second word.

For each starting word A select word B with the maximum statistical probability of the pairs AB (if the probabilities are equal, then select an arbitrary word from the list). As a result, we get 90,959 pairs of words as a unique pairs of words.

For each pair of words AB from the previous line, calculate the frequency of the pair of words (how many times the pair of words occurs in the texts of the School Corpus) – TF(AB) and the inverse document frequency IDF(AB) where: IDF(AB) = ln(n/m) where n = 25 – number of documents and m is the number of documents, containing AB pairs of words in our example among 25 documents.

Multiplying TF(AB) by IDF(AB) produced 90,959 products. Word pairs corresponding to roughly 5 percent of the smallest values of the 90,959 products were declared as stopwords.

As a result, 4548 words are defined as stop words.

The Bigram stopwords are stored in the file stopwords_bigrams.txt, part of the file is pre-sented in Table 3.

**Table 3:** Part of the Uzbek bigram stopwords list, each line starts with the enumerator, following a blank space delimiter and the stopword pair. The English translations of the presented excerpt are: chop etildi – published, har bir – every, kitob jamgarmasi – found of books, nima uchun – why.

Nr.	stopword
…	…
1.	chop etildi
2.	har bir
3.	kitob jamgarmasi
4.	nima uchun
…	…

### 2.4 Collocation method

This method is suggested by the authors of the article. The method is similar to the Bigram method with an important difference: in the bigram method, only the word with the maximum probability is taken as the second word B producing the AB pair. The second word B is only one (if multiple words have the same probability, one is chosen arbitrarily). The collocation method takes all possible words for the second word B, producing additional candidates. This means that using the collocation method more pairs of words were found. The remainder of the method is the same as in the Bigram method. Consider collocation AB words on the total School corpus. There are 489,857 unique pairs of words. Calculate TF(AB) and IDF(AB) as in the bigram method (see Section 1.3). Multiply TF and IDF, creating their product. One pair out of 489,857 unique pairs corresponds to each product. Among 489,857 unique pairs, select 5 percent of pairs whose product is close to zero and declare them as stopwords. Using the Collocation method for the School Corpus, 24,490 pairs of words were detected as stopwords. The Collocation stopwords are stored in the file stopwords_bigrams_with_collocations.txt, part of the file is presented in Table 4.

**Table 4:** Part of the Uzbek collocation stopwords list, each line starts with the enumerator, following a blank space delimiter and the stopword pair. The English translations of the presented excerpt are: uchun darslik – text book for, chop etildi – published, kitob jamg'armasi- found in books.

Nr.	stopword
…	…
5.	uchun darslik
6.	chop etildi
7.	kitob jamgarmasi
……	

## Ethics Statement

The data-gathering process did not involve the use of human subjects. The only possibly problematic ethical aspect comes from the possible privacy concerns of the gathered corpus and consequently the extracted word lists. All the gathered texts are from public school materials that were priory checked and recognised as without any ethical concerns.

## Credit Author Statement

**Khabibulla Madatov:** Conceptualization, Software, Investigation, Supervision, Writing – review & editing; **Shukurla Bekchanov:** Software, Investigation, Writing – review & editing; **Jernej Vičič:** Conceptualization, Investigation, Writing – review & editing, Data Curation.

## Declaration of Competing Interest

The authors declare that they have no known competing financial interests or personal relationships that could have appeared to influence the work reported in this paper.

## Data Availability

Lists of Uzbek stopwords (Original data) (Zenodo).

## Footnotes

1 Uzbek book portal: https://kitob.uz/.
